# Supplementary material for: Genome-Wide Identification of the Soybean LysM-RLK Family Genes and Its Nitrogen Response
Source: Int J Mol Sci. 2023 Sep 3;24(17):13621. doi: 10.3390/ijms241713621 (PMC10487828; doi:10.3390/ijms241713621)
Supplement: Supplementary file 1 [file ijms-24-13621-s001.zip › Supplementary Figures.pdf]

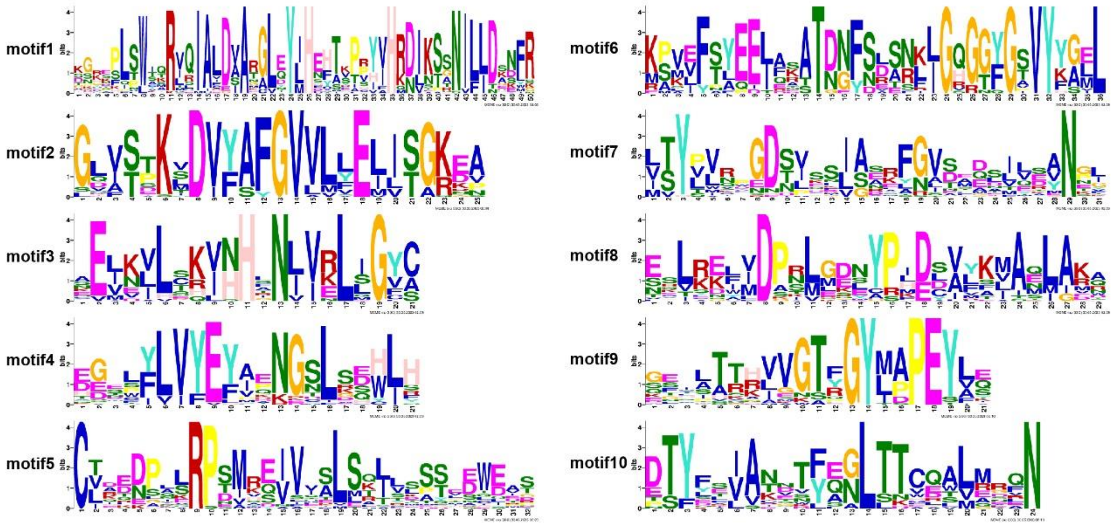

Figure S1. Conversed motif of soybean LysM-RLK amino acid sequences. The conversed motifs of LysM-RLK proteins were analyzed by the MEME online program. The character and size of each logo indicates the relative abundance of each nucleotide in the sequence.

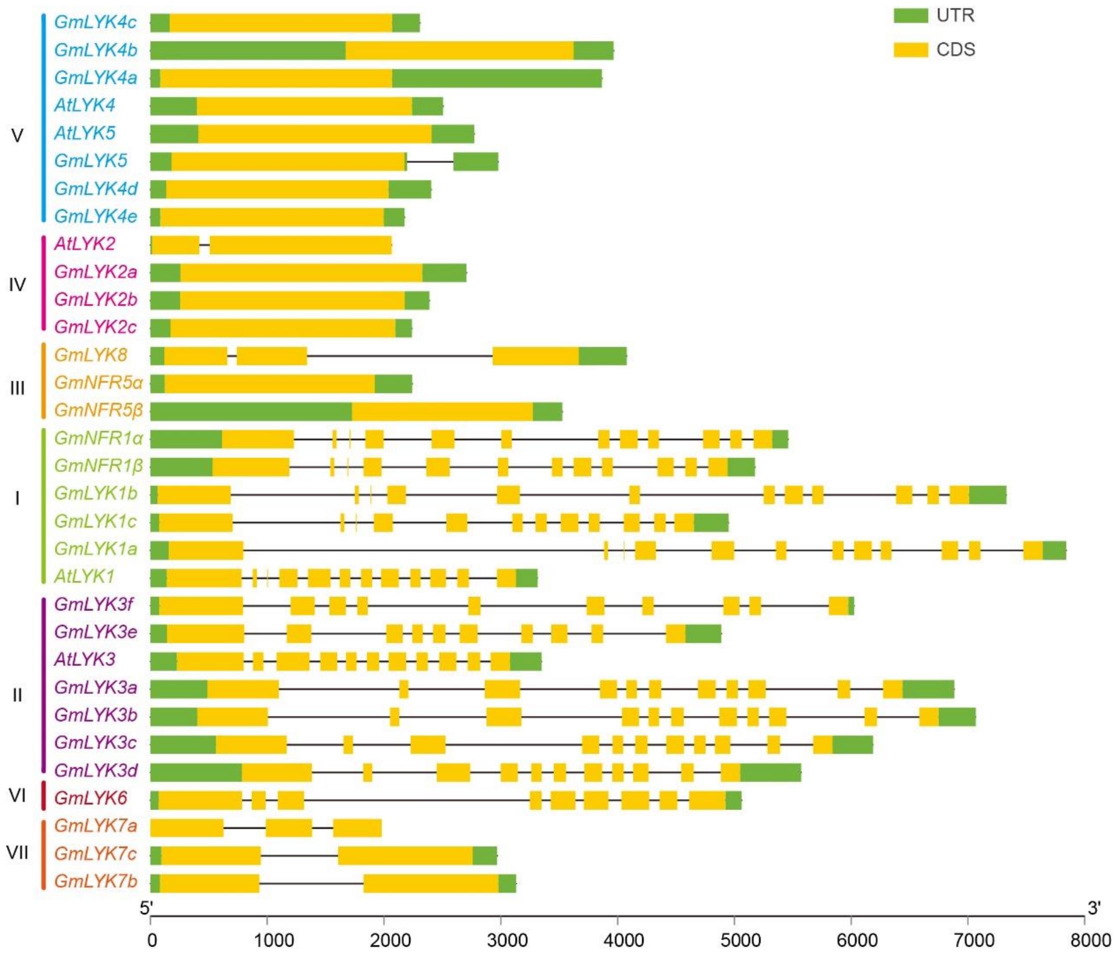

Figure S2. Gene structures of LysM-RLK genes from soybean and Arabidopsis.

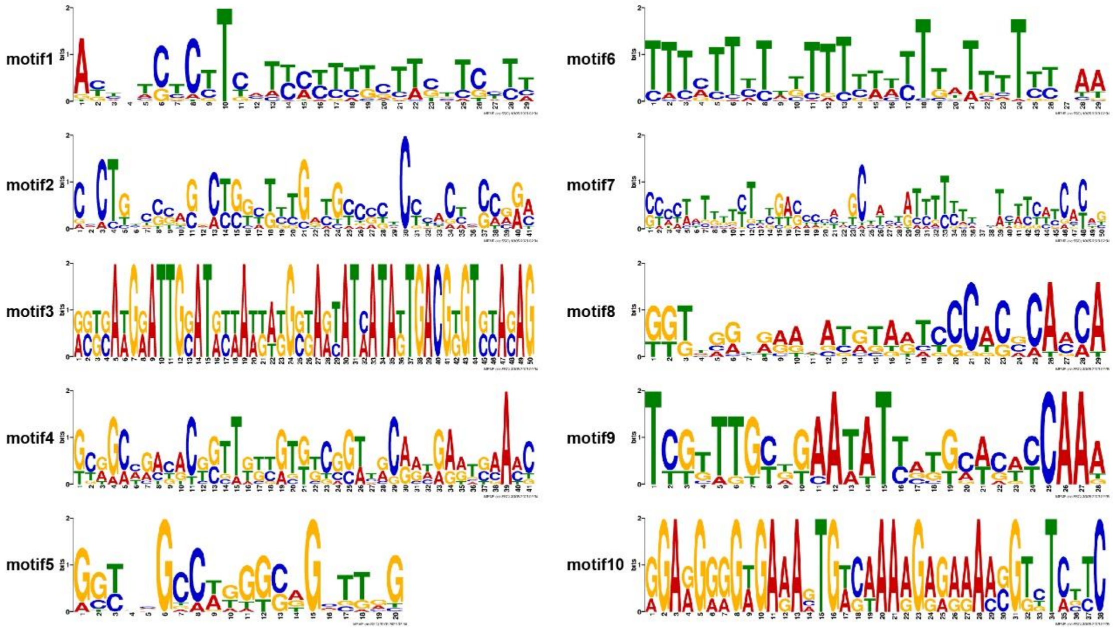

Figure S3. Conserved motif on the promoter sequences of soybean LysM-RLK genes. The conversed motifs identified from the promoters of *LysM-RLKs* were obtained from MEME analysis. The character and size of each logo indicates the relative abundance of each nucleotide in the sequence.
